# Microbiomes of Various Maternal Body Systems Are Predictive of Calf Digestive Bacterial Ecology

**DOI:** 10.3390/ani11082210

**Published:** 2021-07-26

**Authors:** Connor E. Owens, Haley G. Huffard, Alexandra I. Nin-Velez, Jane Duncan, Chrissy L. Teets, Kristy M. Daniels, Alan D. Ealy, Robert E. James, Katharine F. Knowlton, Rebecca R. Cockrum

**Affiliations:** 1 Department of Dairy Science, Virginia Polytechnic Institute and State University, Blacksburg, VA 24061, USA; cowens46@vt.edu (C.E.O.); hhuffard@vt.edu (H.G.H.); aninvele@vt.edu (A.I.N.-V.); aduncan@vt.edu (J.D.); cteets77@vt.edu (C.L.T.); danielsk@vt.edu (K.M.D.); jamesre@vt.edu (R.E.J.); kknowlto@vt.edu (K.F.K.); 2 Department of Animal and Poultry Sciences, Virginia Polytechnic Institute and State University, Blacksburg, VA 24061, USA; ealy@vt.edu

**Keywords:** microbiome, dam, dairy calf

## Abstract

**Simple Summary:**

Composition of the bacterial community in a newborn’s gut plays a role in their early development and immune system function. Understanding relationships between the bacterial communities of cows and their offspring can help identify which communities have a greater influence on bacterial community development. We examined bacteria at various sites of the cow at birth and bacteria in their calf’s gut throughout early life to understand their relationship. We found that bacteria in the cow’s reproductive tract, gut, and even milk all served as predictors for calf gut bacteria from birth up to 60 d old. Further exploration of these relationships as well as examining relationships of these bacterial communities with illness could help to prevent disease in calves.

**Abstract:**

Body systems once thought sterile at birth instead have complex and sometimes abundant microbial ecosystems. However, relationships between dam and calf microbial ecosystems are still unclear. The objectives of this study were to (1) characterize the various maternal and calf microbiomes during peri-partum and post-partum periods and (2) examine the influence of the maternal microbiome on calf fecal microbiome composition during the pre-weaning phase. Multiparous Holstein cows were placed in individual, freshly bedded box stalls 14 d before expected calving. Caudal vaginal fluid samples were collected approximately 24 h before calving and dam fecal, oral, colostrum, and placenta samples were collected immediately after calving. Calf fecal samples were collected at birth (meconium) and 24 h, 7 d, 42 d, and 60 d of age. Amplicons covering V4 16S rDNA regions were generated using DNA extracted from all samples and were sequenced using 300 bp paired end Illumina MiSeq sequencing. Spearman rank correlations were performed between genera in maternal and calf fecal microbiomes. Negative binomial regression models were created for genera in calf fecal samples at each time point using genera in maternal microbiomes. We determined that Bacteroidetes dominated the calf fecal microbiome at all time points (relative abundance ≥42.55%) except for 24 h post-calving, whereas Proteobacteria were the dominant phylum (relative abundance = 85.10%). Maternal fecal, oral, placental, vaginal, and colostrum microbiomes were significant predictors of calf fecal microbiome throughout pre-weaning. Results indicate that calf fecal microbiome inoculation and development may be derived from various maternal sources. Maternal microbiomes could be used to predict calf microbiome development, but further research on the environmental and genetic influences is needed.

## 1. Introduction

Bacterial colonization of the newborn gut during and after parturition influences intestinal development and immune system function [1,2]. Previous studies have demonstrated microbiota in meconium, or calf feces present at birth, are similar to those in feces in calves up to 24 h of age [3]. There is a dramatic shift in the fecal microbiota at 24 h, demonstrating the calf fecal microbial community is influenced very early in life [3]. These early influences can include bacteria from the dam as well as the environment, but the extent of their influence is not yet fully understood. Understanding the sources of colonization and their influence on gut development is key in determining what the “expected” microbiome is, as deviations in the gut microbiome can reflect an animal’s response to environmental or physiological stressors [4,5]. However, the “expected” or “normal” calf gut microbiome is not yet fully understood and may not be the same for every animal.

The dam’s microbiomes from the uterine environment, vaginal canal, feces, saliva, and colostrum are a major influence on calf digestive system microbial colonization. While bacteria in the feces are not fully representative of those in other portions of the gastrointestinal tract, like the rumen, changes in the fecal microbiome have reflected calves’ response to their environment and can predict risk of dysbiosis [6,7,8]. The early calf fecal microbiome is dominated by bacteria present in the vaginal microbiome of the dam, as the vaginal microbiome shared the most bacteria with calf feces from 30 min to 48 h after birth when compared to dam feces or colostrum [9]. Rumen microbiota differed based on mode of birth (vaginal vs. cesarean section), which demonstrates the vaginal canal as a major influence on the entire gastrointestinal tract [10]. Cow feces and colostrum do influence calf digestive development, as both shared abundant bacteria with calf feces during the first 24 h post-partum [10]. There is also evidence that bacteria from these sources influence the microbiome up to 21 d of age [11]. However, many bacteria in calf feces have not been found in dam vaginal, fecal, or colostrum microbiomes. Consumption of bacteria is the most common method of gut inoculation, but bacteria also have the ability to cross into the blood and lymphatic system and could potentially inoculate other systems. The entero-mammary pathway is one proposed mechanism in which bacteria travel from the gut to inoculate the mammary gland [12]. It is unclear if a similar pathway exists for other body systems and what their relationship is with the gut. Characterizing other maternal sources and routes of inoculation might identify the origin of these bacteria and further explain colonization of the calf gut and other systems.

Another potential source of inoculation is the upper reproductive tract of the dam. While previously considered sterile, recent evidence in multiple species has identified microbiomes distinct to locations within the upper reproductive tract and in the fetus itself [13,14,15]. These bacteria could have migrated from the lower reproductive tract or the dam’s gut, as bacteria may cross the intestinal epithelium and travel to the uterus during periods of intestinal hyperpermeability or “leaky gut” [12,16]. The uterus contains a lumenal environment and epithelium distinct from the intestine, which would support its own unique bacterial community and is available to inoculate the calf gut [17,18]. Bacteria have been identified in multiple locations of the pregnant tract of dairy cattle and these additional microbiomes could serve as a source of calf gut colonization [14]. It would be difficult and potentially dangerous to collect samples from the post-partum uterus, but the placenta could be representative of the upper reproductive microbiome. However, potential relationships between the placental and calf microbiomes have yet to be described. Further to this, due to the known influence of other maternal microbiomes, the placenta’s potential influence cannot be studied independently.

Various biological system microbiomes have been linked to health, fertility, and efficiency and have been shown to influence the microbial ecology of other systems. Therefore, environmental or genetic changes in one biological system, such as the digestive system, may unintentionally affect other microbiomes; thus, it is critical to elucidate the interrelationships of these systems within individuals and between mothers and their progeny. The objectives of this study were (1) to characterize the maternal and calf fecal microbiomes during peri-partum and post-partum periods and (2) examine the influence of the maternal microbiome on calf fecal microbiome development during the pre-weaning phase. We hypothesized that dam reproductive, fecal, and colostrum microbiomes would all play significant roles in calf gut colonization.

## 2. Materials and Methods

### 2.1. Animal Observation and Sample Collection

Animal procedures were approved by the Virginia Polytechnic Institute and State University Institutional Animal Care and Use Committee (protocol #17-187-DASC). Multiparous, pregnant Holstein cows (*n* = 13) were enrolled in the study 12 to 14 d prior to expected calving date and housed in individual box stalls. Box stalls were bedded with sawdust and re-bedded after each calving to avoid contamination across dams. Close-up dry cows were fed a total mixed ration twice daily at 0900 h and 1900 h and were provided ad libitum access to water. The Moocall calving alert system sensor (Moocall Ltd., Dublin, Ireland) was placed on the dam’s tail 7 d prior to expected calving to alert when calving began. Sterile, flocked swabs (Puritan, Guilford, ME, USA) were used to sample vaginal fluid from the dam’s caudal vagina within 24 h prior to parturition and snap frozen in cryotubes using liquid nitrogen.

At parturition, calves (*n* = 13; bulls = 9, heifers = 4) were immediately separated from dams and transferred to a clean 111.28 × 55.40 × 46.13 cm^3^ plastic container containing fresh wood shavings to prevent environmental contact. The container was rebedded between each calving. Calves were weighed immediately after birth. Sterile, flocked swabs (Puritan, Guilford, ME, USA) were used to collect meconium from newborn calves before passage out of the body and oral samples from the left and right buccal wall of the dam immediately after parturition. These samples were snap frozen in cryotubes using liquid nitrogen. Dam fecal samples were removed from the rectum using a clean palpation sleeve and sterile flocked swabs were used to collect samples before being snap frozen in cryotubes using liquid nitrogen.

Representative colostrum samples were aseptically collected before milking and frozen at −20 °C. Remaining colostrum was collected using a stainless-steel portable bucket milking machine. Colostrum was required to have a Brix score ≥22% using a Brix refractometer (VEE GEE Scientific, Vernon Hills, IL, USA), which correlates to ≥50 g/L of immunoglobulin G (IgG) in the colostrum. If colostrum did not achieve a Brix score ≥22%, the dam–calf pair were removed from the study. One dam and one bull calf were removed due to failure to meet colostrum requirements. Calves were assigned individual bottles and nipples at colostrum feeding to be used for the remainder of the study. Calves were bottle fed 4 L of their dam’s colostrum within 1 h post-birth. Antibiotics used to treat common bacteria-associated calf morbidities might influence gut microbiome composition. In order to mitigate the use of antibiotics in the study, calves were bottle fed an additional 2 L of colostrum at 12 h post-calving.

Sections of placenta were collected within 6 h post-birth after passage through the vagina but before coming in contact with the ground using a sterile scalpel. Cotyledon tissue was snap frozen in cryotubes using liquid nitrogen.

After their initial colostrum feeding, calves were moved to individual, sawdust-bedded hutches and remained there through the end of the study. Sterile, flocked swabs (Puritan, Guilford, ME, USA) were used to collect calf fecal samples at 24 h post birth. Blood was collected from each calf 24 h post birth via jugular venipuncture using Monoject blood tubes with no additive (Covidien, Mansfield, MA, USA). Blood was stored at 4 °C for 12 h and then centrifuged at 2000× *g* for 20 min at 4 °C to isolate serum.

Calves were fed 4 L of 27.0% CP, 20.0% fat milk replacer (Cow’s Match^®^ ColdFront^®^ Medicated (67 mg/kg lasalocid sodium), Land O’Lakes^®^ Animal Milk Products Co., Shoreview, MN, USA) twice daily at 600 h and 1800 h beginning approximately 24 h post-birth. Calves were fed using individually-assigned bottles and nipples to avoid cross-contamination. Calves were allowed ad libitum access to water at 1 d of age. All calves were vaccinated with INFORCE™ 3 (Bovine Rhinotracheitis, Parainfluenza 3, Bovine Respiratory Syncytial Virus Vaccine; 2 mL intranasally; Zoetis Inc., Kalamazoo, MI, USA) at 4 d of age. At 28 d of age, calves were given ad libitum access to a 22% CP starter grain (Intensity 22% Textured Calf Starter Medicated, Cargill Animal Nutrition, MN, USA). Step down weaning began at 42 d of age, with calves fed 3 L of milk replacer twice daily from 42 to 49 d and 2 L of milk replacer twice daily from 50 to 56 d. Calves were completely weaned at 57 d and removed from the study at 60 d. Water and starter refusals were measured at each feeding. Calves were observed at each feeding for symptoms of scours. Calves were weighed weekly approximately 1 h prior to evening feeding. Sterile, flocked swabs (Puritan, Guilford, ME, USA) were used to collect calf fecal samples at 7 d, 42 d, and 60 d.

### 2.2. Serum and Colostrum IgG

Colostrum and calf serum IgG concentrations were measured using a commercial Bovine IgG ELISA (Bethyl Laboratories, Inc., Montgomery, TX, USA) according to the manufacturer’s protocol in order to confirm successful passive transfer (serum IgG ≥ 1000 mg/dL). Plates were read at 450 nm (BioTek Instruments, Winooski, VT, USA) and data were analyzed using a four-parameter logistic curve software (MyAssays Ltd., Brighton, UK). Samples with an intra assay CV of <10% and inter assay CV of <15% were used to determine IgG concentration.

### 2.3. DNA Extraction and Sequencing

The mis-estimation of calving events led to incomplete sample sets from eight of the cow–calf pairs. Of these eight, six cow–calf pairs were removed from the study because they were missing two or more key pre-calving microbial samples, while two cow–calf pairs yielded nearly complete sample sets, only missing pre-birth vaginal samples. One dam and one bull calf were removed due to failure to meet colostrum requirements. This left the study with six cow–calf pairs for microbiome analyses, with three heifers and three bull calves.

Bacterial DNA was extracted from all oral, fecal, and vaginal swab samples using the QIAamp BiOstic Bacteremia DNA kit (Qiagen, Germantown, MD, USA). Bacterial DNA was extracted from placenta and colostrum samples using the Qiagen Mini Stool Kit (Qiagen, Germantown, MD, USA). Colostrum was initially centrifuged at 12,000× *g* for 30 min at 4 °C in order to pellet bacteria before DNA extraction. Before DNA precipitation, each sample was treated with 20 µg RNAse A at room temperature for 3 min to remove any potential RNA contamination. Qubit 2.0 Fluorometer and Qubit dsDNA HS Assay kit (Invitrogen, Carlsbad, CA, USA) were used to measure DNA quality and quantity before sequencing.

Samples were submitted to the Virginia Bioinformatics Institute Genomics Research Laboratory (Blacksburg, VA, USA) for library preparation and sequencing. 16S rDNA amplicons covering variable region V4 were generated using primers 515F–806R (reverse barcoded: FWD: GTGCCAGCMGCCGCGGTAA; REV: GGACTACHVGGGTWTCTAAT) [19]. Amplicons were pooled and purified using a Pippin Prep 1.5% gel cassette (Sage Science, Inc., Beverly, MA, USA). Amplicon libraries were sequenced using 300 bp paired end sequencing via Illumina MiSeq (Illumina, San Diego, CA, USA).

### 2.4. Bioinformatics Analysis

#### 2.4.1. Taxonomic Profiling

Taxonomic profiling was performed using CLC Genomics Workbench Microbial Genomics Module version 12.0 (Qiagen, Germantown, MD, USA). Amplicon sequences had adapters removed and were filtered to remove reads with a Phred score <30. Filtered reads were aligned to the 97% Greengenes database version 13.8 to be separated into operational taxonomic units (**OTU**). These OTU were aligned using Multiple Sequence Comparison by Log-Expectation (MUSCLE, version 3.8.31) [20] with a maximum of 16 iterations and a minimum combined abundance of 10 across samples. A phylogenetic tree was constructed using aligned OTU with a Neighbor Joining method, General Time Reversible nucleotide substitution model, and Whelan and Goldman (WAG) protein substitution model [21,22,23].

#### 2.4.2. Alpha and Beta Diversity

Alpha diversity, the microbial diversity within a sample, was measured using Shannon entropy, Simpson’s index, and phylogenetic diversity (*PD*) based on the constructed phylogenetic tree.

$$PD=\overset{n}{\underset{i=1}{\sum }}b_{i\;}I(p_{i}>0)$$

where *n* was the number of branches within the phylogenetic tree, *b_i_* was the length of branch *I*, *p_i_* was proportion of taxa descending from branch *i*, and the *I* (*p_i_ >* 0) assumed the value of 1 if any taxa descending from branch *i* were present in the sample or 0 otherwise. A Kruskal–Wallis H test was used to measure differences in alpha diversity measures based on sample type. A *p*-value ≤ 0.05 was considered significant.

Beta diversity, diversity in microbial community structure between samples, was measured using weighted Unifrac distances (*d*^(*W*)^) based on the constructed phylogenetic tree.

$$d^{\left(W\right)}=\frac{\sum_{i=1}^{n}b_{i}\left|p_{i}^{A}-p_{i}^{B}\right|}{\sum_{i=1}^{n}b_{i}\left(p_{i}^{A}+p_{i}^{B}\right)}$$

where *n* was the number of branches in the phylogenetic tree, *b_i_* was the branch length *i*, and *p_i_^A^* and *p_i_^B^* were the proportion of taxa descending from branch *i* in samples *A* and *B*. A permutational multivariate analysis of variance (PERMANOVA) was used to measure difference in Beta diversity based on the main effects of sample type and calf sex [24]. A *p*-value ≤ 0.05 was considered significant. A Bonferroni *p*-value ≤ 0.05 was considered significant when multiple pair-wise comparisons were made between various sample types.

#### 2.4.3. Microbiome Associations

Spearman ranked correlations were performed among maternal microbiomes, among each calf fecal microbiomes, and between maternal microbiomes and calf fecal microbiomes based on genera relative abundance using cor.test function in the package stats in R version 3.6.1 [25]. A *p*-value ≤ 0.05 was considered significant.

A negative binomial regression model was created using genera count data to evaluate the ability of dam’s placental, colostrum, vaginal, fecal, and oral microbiomes to predict calf fecal microbiomes at each timepoint. The following model was created in R version 3.6.1 [25] and the glm.nb function within the MASS package version 7.3-51.5 [26]:

$$\ln\mu =\beta_{0}+\beta_{1}x_{1}+\beta_{2}x_{2}+\beta_{3}x_{3}+\beta_{4}x_{4}+\beta_{5}x_{5}$$

where *μ* is calf fecal bacteria count at a given time point, *β*_0_ is the intercept, *x*_1_*–x*_5_ are the dam placental, colostrum, vaginal, oral, and fecal bacteria count, respectively, and *β*_1_–*β*_5_ are the expected change in ln *μ* if *x_i_* changes by 1. Maternal microbiome predictors were considered significant if *p* ≤ 0.05.

## 3. Results

### 3.1. Descriptive Statistics

Twelve dams gave birth to calves that met our criteria (bulls = 8, heifers = 4; Table 1). There were no signs of dystocia and calvings did not require assistance. Serum IgG concentrations indicated successful passive transfer of immunity in all calves (calf serum IgG = 2997 ± 251 mg/dL, Table 1). Calves had no signs of scouring or illness during the experiment.

### 3.2. Bioinformatics Analyses

**Figure 1:**
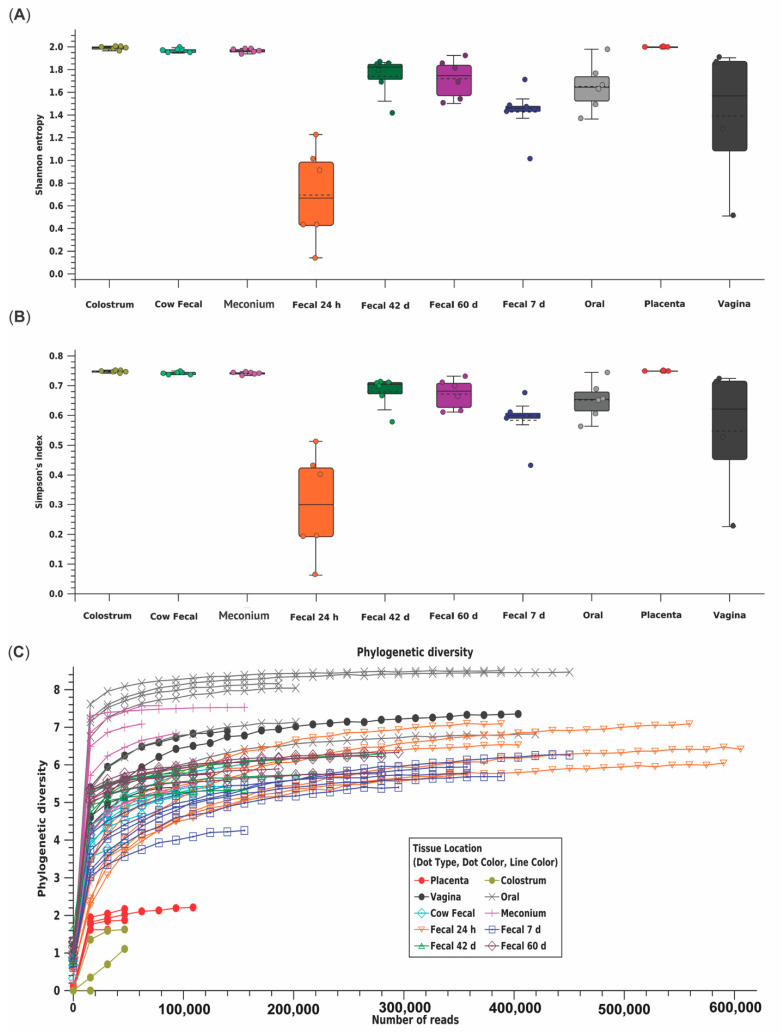
Alpha diversity, or microbial diversity within a sample, as measured by (**A**) Shannon entropy, (**B**) Simpson’s index, and (**C**) phylogenetic diversity for each maternal (placenta, colostrum, oral, fecal, *n* = 6; vaginal, *n* = 4) and calf (meconium, 24 h, 7d, 42 d, and 60 d fecal; *n* = 6) microbiome sample type from multiparous Holstein cow–calf pairs. Calf feces at 24 h had reduced diversity compared to other calf feces timepoints using Shannon entropy and Simpson’s index. Placenta and colostrum had lower phylogenetic diversity than other maternal and calf microbiome sample types.

**Figure 2:**
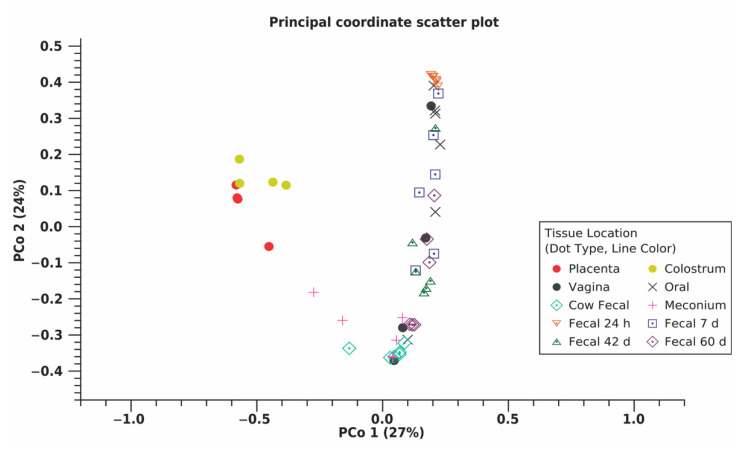
Principle coordinate (PCo) scatter plot using beta diversity or microbial diversity between samples, as measured by weighted Unifrac distances for each maternal (placenta, colostrum, oral, fecal, *n* = 6; vaginal, *n* = 4) and calf (meconium, 24 h, 7d, 42 d, and 60 d fecal; *n* = 6) microbiome sample type from multiparous Holstein cow–calf pairs. A permutational multivariate analysis of variance found there was a difference in beta diversity based on sample type (*p* < 0.001). Placenta and colostrum clustered independently from other sample types, potentially due to low phylogenetic diversity within these samples. Meconium and dam fecal samples clustered closely. Clustering of fecal samples from 24 h to 60 d indicated calf fecal samples became more similar to dam fecal samples over time.

Across all samples, a total of 18,852 OTU were identified using 11,777,504 reads (Table 2). Shannon entropy and Simpson’s index indicated the 24 h calf fecal microbiome had reduced diversity compared to other calf fecal microbiomes [(*p* ≤0.010) (Figure 1A,B, Table S1)]. Placenta and colostrum had low phylogenetic diversity compared to other dam and calf microbiomes (Figure 1C). Beta diversity indicated placenta and colostrum samples clustered independently from other samples (Figure 2). There was a difference in beta diversity based on sample type (*p* < 0.001), but further pairwise comparisons did not indicate a difference between specific sample types (*p* ≥ 0.097; Table S2). No difference in beta diversity was observed based on calf sex (*p* = 0.842).

**Figure 3:**
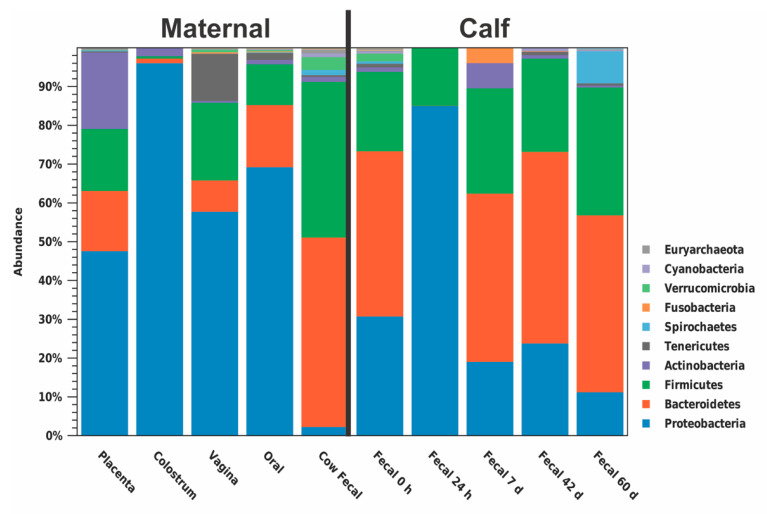
Relative abundance of phyla in each multiparous Holstein maternal (placenta, colostrum, oral, fecal, *n* = 6; vaginal, *n* = 4) and calf (meconium, 24 h, 7d, 42 d, and 60 d fecal; *n* = 6) sample type. Proteobacteria were the dominant phylum within placenta, vagina, colostrum, oral, and 24 h calf fecal samples, while Bacteroides were the dominant phylum in dam fecal, meconium, 7 d, 42 d, and 60 d calf fecal samples.

**Figure 4:**
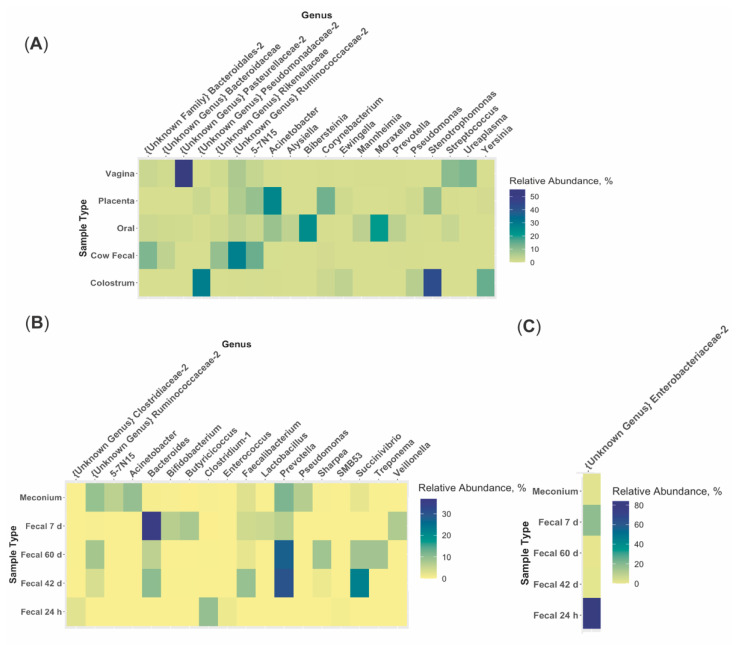
Relative abundance of the 20 most abundant genera within (**A**) each multiparous Holstein maternal (placenta, colostrum, oral, fecal, *n* = 6; vaginal, *n* = 4) and (**B**,**C**) calf (meconium, 24 h, 7d, 42 d, and 60 d fecal; *n* = 6) sample type. Note that the scale in each figure varies in order to appropriately capture variation in genera between sample types. Maternal sample types greatly varied from one another at the genus level. No one genus in meconium samples had a relative abundance >11.56%. An unidentified genus within the family *Enterobacteriaceae* dominated the 24 h fecal microbiome (relative abundance = 83.94%). Calf 42 d and 60 d fecal samples were the only samples with similar dominant genera (*Prevotella*, relative abundance = 30.23% and 27.83%, respectively).

The predominant phylum in colostrum, placenta, vagina, dam oral, and calf 24 h fecal samples was Proteobacteria (96.15%, 47.70%, 57.84%, 69.33%, and 85.10%, respectively; Figure 3, Table S3). The predominant phylum in dam fecal, meconium, calf 7 d, 42 d, and 60d fecal samples was Bacteroidetes (48.81%, 42.55%, 43.36%, 49.35%, and 45.58%, respectively; Figure 3). At the genera level, no one genus was dominant across all maternal or calf sample types (Figure 4; Table S4). An unidentified genus within the family *Pasteurellaceae* dominated the vaginal microbiome (55.31%) and *Stenotrophomonas* dominated the colostrum microbiome (42.72%; Figure 4A). Other maternal microbiomes did not contain one genus with a relative abundance >27.98%. Meconium, 42 d calf fecal, and 60 d calf fecal all had *Prevotella* as the most abundant genus (11.56%, 30.23%, and 27.83%, respectively; Figure 4B). The 24 h calf fecal sample was dominated by an unknown genus in the family *Enterobacteriaceae* (83.94%; Figure 4C).

### 3.3. Microbiome Associations

Spearman ranked correlations were performed among maternal microbiomes, among calf fecal microbiomes, and between maternal microbiomes and calf fecal microbiomes using genera relative abundance. All correlations were significant (*p* ≤ 0.001). The dam fecal microbiome had a moderate correlation with the vaginal microbiome (Table 3). From 24 h to 60 d, there was moderate to strong correlation between calf fecal microbiomes of subsequent timepoints (Table 3). Correlations between calf fecal microbiomes and dam and vaginal microbiomes increased with age (Table 3).

Negative binomial regression models were created to estimate predictive ability of maternal microbiomes on calf fecal microbiomes (Table 4). Each maternal microbiome was a significant predictor for at least two time points. None of the maternal microbiomes were significant predictors for all calf fecal microbiomes.

## 4. Discussion

The objectives of this study were to characterize the various maternal and calf fecal microbiomes during the peri-partum and post-partum periods and examine the relationship of the maternal microbiome with calf fecal microbiome development during the pre-weaning phase. Using 16S amplicon sequencing, we identified unique microbiomes within the dam’s placenta, vagina, colostrum, feces, and oral cavity and the calves’ feces. Genera in the dam oral microbiome had a moderate positive correlation with genera in the early calf fecal microbiomes. All maternal microbiomes were a significant predictor for the calf microbiome during at least 2 time points during pre-weaning. No maternal microbiome was a significant predictor at every time point.

Inoculation of the calf microbiome can stem from many sources; (1) the dam before and during birth, (2) the diet, and (3) the environment. Previous research has investigated the influence of the dam on early rumen or intestinal inoculation (birth to 7 d) or exclusively diet on rumen microbiome development in dairy calves [11,27,28]. Our study was the first aimed at associating how the maternal microbiomes, including placental, vaginal, colostrum, oral, and fecal, are related to the calf gut microbial development throughout the pre-weaning phase (birth to 60 d).

### 4.1. Early Changes in the Calf Fecal Microbiome

Dramatic changes occur in the neonatal calf fecal microbiome between birth and 24 h of age. In the current study, the fecal microbiome at 24 h of age was almost entirely made up of Proteobacteria, compared to meconium collected at birth, which mainly comprised Bacteroidetes, Proteobacteria, and Firmicutes. The 24 h fecal microbiome also had a reduction in alpha diversity measures compared to meconium, indicating reduced diversity in the microbial community structure. This has been seen previously in a recent study investigating the composition of the perinatal intestinal microbiome in Holstein and Ayrshire calves [27]. Others have observed the neonatal gut microbiota as an unstable community due to its rapid variation and colonization by facultative anaerobes, specifically Proteobacteria [29]. Proteobacteria play an important role in preparing the neonatal gut microbiota for successive colonization by strict anaerobes by consuming oxygen, altering pH, lowering redox potential, and producing carbon dioxide and nutrients [29,30,31]. Proteobacteria have been observed as a dominant phylum in many environmental niches, including soil [32], plants [33], freshwater [34], seawater [35], and the atmosphere [29,36], suggesting that the high prevalence of Proteobacteria in the fecal microbiome of calves at 24 h of age could be a result of the calf’s first environmental exposure. This suggests significant environmental effects on calf gut microbiome within a short period of time.

The most abundant genera in the young calf fecal microbiome may play a role in microbiome composition and calf response to disease. *Prevotella* and an unclassified *Enterobacteriaceae* genus were the most abundant genera in calf feces from during the first 24 h after birth. In mice, increased abundance of some species of *Prevotella* led to decreased acetate and increased butyrate in the large intestine and increased production of inflammatory cytokines [37]. In calves, gut inflammation followed by prolonged dysbiosis caused by *Enterobacteriaceae* has resulted in calf diarrhea [38]. It is possible that increased abundance of these genera could alter the newborn gut microenvironment and exacerbate calf illness. Further research examining specific inclusion and exclusion of these microbes alongside immune response measurements could elucidate host–microbe interactions in the calf gut.

### 4.2. Variation between Maternal Sources

The current study examined how bacteria from various maternal sites inoculated the calf gut, but one important aspect not within the scope of this study was how those maternal microbiomes were initially inoculated. Consumption of bacteria and passage from the oral cavity through the gastrointestinal tract could explain inoculation of the gut microbiome and its development, but it does not account for the colostrum or reproductive tract microbiomes. One potential method of bacterial colonization of these sites is through an entero-mammary pathway. In this proposed pathway, bacteria in the maternal intestine permeate the intestinal epithelium and enter the lymphatic or circulatory system, allowing them to travel to the mammary gland or reproductive tract [12]. There is little evidence in the dairy cow demonstrating the existence of this pathway, but common gut bacteria like *Ruminococcus* and *Bifidobacterium* have been identified in mammary secretions, blood, and feces within the same dam [16]. In mice, *Enterococcus*, *Streptococcus*, *Staphylococcus*, and *Propionibacterium* were cultured from umbilical cord blood [39]. Additionally, pregnant mice were orally inoculated with genetically labeled *Enterobacterium faecium* that was then identified in amniotic fluid [39]. However, the murine placenta is very different in both structure and transport function from the ruminant placenta [40,41,42]. Further research in dairy cattle using similar labeled bacteria methods would be needed to support the existence of this pathway and explain how these maternal sites are inoculated.

The microbial composition and community structure between maternal sites provides some insight on how these various sites are related and could potentially influence each other. In the current study, dam vaginal, oral, and fecal microbiomes were moderately correlated with one another. However, the principal coordinate scatter plot demonstrated dam fecal samples tightly clustered while vaginal and oral samples were not. Previous literature shows similar results and provides some insight on how various maternal microbiomes are inoculated, but these results also point towards the difficulty of determining bacterial contamination versus inoculant [27,43]. The broader clustering of the vaginal and oral microbiomes makes sense, as these sites not only contain bacteria typically commensal to that location, but they are also consistently exposed to sources of new bacteria, like feed for the oral cavity or feces and bedding for the vagina, which would increase beta diversity of the microbiome [27,44]. Compared to the oral cavity or vagina, the cow’s colon is exposed to fewer external sources of bacteria; therefore, microbial diversity between fecal samples is expected to be reduced.

Similarly, sections of the reproductive tract, like the vagina, cervix, uterus, and oviduct, support the growth of specific subsets of bacteria, but the microbial composition and diversity of each section could be influenced by those adjacent to it [45,46]. We observed a moderate correlation between the placental and vaginal microbiomes, but we also observed lower phylogenetic diversity in placental samples compared to vaginal samples and separate clustering of placental and vaginal samples in the principal coordinate scatter plot. This is expected, as bacteria in the vagina may enter the uterus throughout pregnancy, but the difference between vaginal and uterine/placental environments might support the abundance of certain bacteria over others [17,18,47]. There is still the possibility that bacteria utilize a pathway similar to the entero-mammary axis to reach the uterus or that some bacteria found in the placenta are contaminants from the vagina. Research utilizing both fluorescent in situ hybridization (FISH) and 16S amplicon sequencing on multiple samples throughout the pregnant reproductive tract could provide insight into the route bacteria use to colonize the uterus, if certain bacteria have a location preference within the reproductive tract, and help differentiate between commensal and contaminant bacteria.

### 4.3. Microbiome Heritability

One potential component that shapes microbiomes which we were unable to account for is heritability. A core rumen microbiome has been identified in beef and dairy cattle with an estimated narrow sense heritability of ≥0.15 [48,49]. This heritable subset of rumen bacteria has also been associated with feed efficiency and methane emissions [50]. This could mean the fecal microbiome is also heritable and could influence cow performance. However, a much larger study examining various maternal sources of calf fecal bacteria are needed to estimate their heritability.

Instead of direct passage from parent to progeny, microbiome heritability may be due to genetic influence on tissue morphology. Which taxa dominate a particular location is influenced by the available proteins, metabolites, and molecular substrates, as certain bacteria are more efficient at surviving in a particular environment than others [17,18,47]. In an animal’s body, organ luminal environment influences and is influenced by tissue morphology, including type of cells, abundance of each type, and level of activity within these cells [51]. In humans, genomic markers have been associated with tissue morphology, including skeletal muscle, pancreas, and reproductive tissues [52]. This genetic influence on morphology would then influence the tissue environment and subsequently the microbiome. We observed in the principal coordinate scatter plot that samples were clustered based on location within the dam’s body, with placenta, vagina, oral cavity, udder, and large intestine all having distinct morphology. It is possible there is a genetic influence on this morphology and the microbiomes of each sample type, but further research is needed to examine the genetic influence on morphology in cattle as well as its association with the various microbiomes.

### 4.4. Study Limitations and Future Directions

Each sample type from the dam seems to predict the calf fecal microbiome during at least two time points and no one maternal microbiome seems to be the sole influencer of calf fecal development. This supports our hypothesis that each maternal microbiome plays some role in calf gut inoculation and development. However, our sample size was limited to only six calf–dam pairs. Additionally, it is likely that the neonatal calf microbiome is influenced by the birth environment. The neonatal calf is born with a diverse microbiome that is immediately subject to rapid changes due to exposure to the environment [27]. One limitation of our study is the unknown microbial community within the birth environment (calving pen) and housing environment (calf hutches). All cows were calved in stalls bedded with fresh shavings three to four days prior to birth, but the degree of fecal, aerial, or other bacterial contamination in that short pre-calving window certainly varied. Additionally, bacteria in the water made available to the calves and within each of the calves’ bottles would contribute to development of the calf gut microbiome. Research in pigs has demonstrated bacteria acquired from the environment can influence microbial composition at the gut surface [53]. We attempted to limit potential environmental contamination during and following parturition, but analyzing the microbiome from environmental sources like water or bedding would allow separation of source contributions to the calf microbiome. Another limitation to our study was that all animals were from the same location. This removes variation due to location, but identifying a core maternal microbiome that is the main influence for calf microbial development may require calf–dam pairs from various locations to account for this. Future studies with increased sample size and accounting for further sources of environmental variation would support mathematical modelling to predict calf microbiome development.

## 5. Conclusions

The current study supports our hypothesis that maternal microbiomes, including fecal, oral, colostrum, and reproductive microbiomes, play a role in the calf gut microbiome inoculation and development. All dam microbiomes measured were predictive of the calf fecal microbiome through the preweaning phase, with dam fecal and oral microbiomes having the largest correlation. Studies further validating relationships between these microbiomes as well as other maternal or calf microbiomes are necessary in order to use these microbiomes as a tool for monitoring calf response to environmental stressors.

## Figures and Tables

**Table 1:** Descriptive statistics for Holstein calves and colostrum ^1^ during experiment.

Variable	Mean	SEM	Min	Max
Birth weight, kg	46.00	0.94	43.00	48.00
ADG, kg/d	0.70	0.03	0.61	0.76
Water intake, kg/d	0.60	0.06	0.41	0.82
Feed intake, kg/d	0.61	0.02	0.54	0.68
Fecal score, 0–3 ^2^	0.50	0.00	0.25	0.75
Colostrum brix, % ^3^	26	0.88	23	29
Colostrum volume, L	6.5	0.86	3.8	9.5
Colostrum IgG, mg/dL	13,502	1976	5696	18,570
Calf serum IgG, mg/dL ^4^	2997	251	1783	4388

^1^ Colostrum was collected within 1 h of parturition using a stainless-steel portable bucket milking machine; ^2^ Fecal scores ranged from 0 (normal, solid feces) to 3 (water stool that sifts through bedding) according to the Univeristy of Wisconsin Madision School of Veterinary Medicine’s Calf Health Scoring Chart; ^3^ Colostrum Brix score was measured using a Brix refractometer (VEE GEE Scientific, Vernon Hills, IL, USA); ^4^ Calf serum was collected 24 h after colostrum feeding via jugular venipuncture using Monoject blood tubes with no additive (Covidien, Dublin, Ireland).

**Table 2:** Results from 300 bp paired end sequencing of V4 region of 16S rDNA amplicons on the Illumina MiSeq platform. Results are separated based on type of sample.

Tissue	Total Reads	Reads in OTU ^1^	Number of OTU ^1^
Placenta	385,350 ± 26,534	61,688 ± 15,016	187.00 ± 56.44
Colostrum	362,749 ± 49,633	32,776 ± 6923	20.50 ± 7.42
Vagina	314,095 ± 57,108	184,739 ± 74,332	1436.25 ± 114.11
Oral	431,700 ± 58,540	313,742 ± 50,289	2202.67 ± 466.09
Dam Fecal	204,011 ± 29,443	110,857 ± 16,556	1498.83 ± 176.39
Meconium	244,533 ± 16,662	107,453 ± 14,227	1223.33 ± 146.94
24 h Fecal	543,118 ± 51,403	490,960 ± 44,588	339.33 ± 29.01
7 d Fecal	372,971 ± 44,982	338,514 ± 41,614	406.00 ± 35.87
42 d Fecal	208,827 ± 29,414	176,874 ± 27,221	797.33 ± 27.06
60 d Fecal	262,002 ± 40,738	216,843 ± 33,375	1063.83 ± 95.34

^1^ Operational taxonomic units.

**Table 3:** Spearman rank correlation *r_s_* between genus relative abundance of the maternal microbiomes at calving and its calf’s fecal microbiome from calving until 60 d of age ^1^.

	Placenta	Vagina ^2^	Oral	Fecal	Meconium	24 h	7 d	42 d	60 d
Colostrum	0.175	0.121	0.110	0.056	0.128	0.097	0.073	0.052	0.050
Placenta		0.306	0.292	0.228	0.267	0.221	0.204	0.193	0.210
Vagina ^2^			0.463	0.506	0.142	0.312	0.309	0.329	0.404
Oral				0.432	0.527	0.310	0.309	0.319	0.347
Fecal					0.337	0.329	0.335	0.420	0.477
Meconium						0.276	0.282	0.347	0.360
24 h							0.632	0.402	0.410
7 d								0.523	0.473
42 d									0.729

^1^ Microbiomes were from multiparous Holstein cow–calf pairs (*n* = 6). All correlations had a *p*-value < 0.001; ^2^ Vaginal canal samples were only obtained from 4 Holstein cow–calf pairs.

**Table 4:** Coefficient estimates for negative binomial regression models between maternal microbiomes and calf fecal microbiomes from multiparous Holstein cow–calf pairs (*n* = 6) ^1^.

Maternal Location	Meconium		24 h Fecal		7 d Fecal		42 d Fecal		60 d Fecal	
	Estimate ^3^	*p*	Estimate	*p*	Estimate	*p*	Estimate	*p*	Estimate	*p*
Placenta	4.96 × 10^−4^	0.390	1.87 × 10^−2^	<0.001	−3.02 × 10^−3^	<0.001	−1.75 × 10^−3^	0.27	1.72 × 10^−4^	0.722
Colostrum	2.72 × 10^−3^	<0.001	−4.26 × 10^−3^	<0.001	8.58 × 10^−2^	0.001	−1.81 × 10^−2^	0.180	−2.30 × 10^−3^	0.003
Vagina ^2^	1.23 × 10^−5^	0.130	−2.29 × 10^−6^	0.770	−1.79 × 10^−5^	0.025	−2.93 × 10^−5^	0.180	−1.70 × 10^−5^	0.014
Oral	1.73 × 10^−4^	<0.001	−7.70 × 10^−6^	0.100	3.31 × 10^−5^	<0.001	5.57 × 10^−4^	<0.001	2.78 × 10^−5^	<0.001
Fecal	1.59 × 10^−4^	<0.001	−5.90 × 10^−5^	<0.001	−2.34 × 10^−6^	0.870	4.78 × 10^−5^	0.201	1.19 × 10^−5^	0.312

^1^ Models were created using genera count data. Maternal predictors were considered significant when *p* ≤ 0.05; ^2^ Vaginal canal samples were only obtained from 4 Holstein cow–calf pairs; ^3^ Estimate is the expected change in the natural logarithm of genus read count in the calf microbiome if the genus increases by 1 read in the specified maternal microbiome and genus read count in other maternal microbiomes is held constant.

## Data Availability

The data presented in this study are available on request from the corresponding author.

## Supplementary Materials

The following are available online at https://www.mdpi.com/article/10.3390/ani11082210/s1, Table S1: Kruskal–Wallis H test pairwise results comparing alpha diversity measures based on sample type; Table S2: Permutational Analysis of Variance (PERMANOVA) pairwise comparison results based on sample type using Weighted Unifrac distances; Table S3: Relative abundance of phyla within microbiome of each sample type from multiparous Holstein cows and their calves; Table S4: Relative abundance of genera within microbiomes of each sample type from multiparous Holstein cows and their calves.
