# Supplementary material for: Microbiomes of Various Maternal Body Systems Are Predictive of Calf Digestive Bacterial Ecology
Source: Animals (Basel). 2021 Jul 26;11(8):2210. doi: 10.3390/ani11082210 (PMC8388428; doi:10.3390/ani11082210)
Supplement: Supplementary file 1 [file animals-11-02210-s001.zip › Supplemental Table S1-S3.pdf]

SUPPLEMENTAL INFORMATION

Table S1 Kruskal-Wallis H test pairwise results based on sample type using alpha diversity measures Shannon entropy and Simpson's Index.

Sample Type 1	Sample Type 2	Shannon Entropy *P* – value[1]	Simpson's Index *P* – value[1]
Placenta	Colostrum	0.500	0.500
Placenta	Vagina	0.010	0.010
Placenta	Oral	0.006	0.006
Placenta	Cow Fecal	0.008	0.008
Placenta	Meconium	0.006	0.006
Placenta	24 h Fecal	0.006	0.006
Placenta	7 d Fecal	0.006	0.006
Placenta	42 d Fecal	0.006	0.006
Placenta	60 d Fecal	0.006	0.006
Colostrum	Vagina	0.010	0.010
Colostrum	Oral	0.006	0.006
Colostrum	Cow Fecal	0.050	0.050
Colostrum	Meconium	0.020	0.020
Colostrum	24 h Fecal	0.004	0.004
Colostrum	7 d Fecal	0.004	0.004
Colostrum	42 d Fecal	0.004	0.004
Colostrum	60 d Fecal	0.004	0.004
Vagina	Oral	0.700	0.700
Vagina	Cow Fecal	0.010	0.010
Vagina	Meconium	0.010	0.010
Vagina	24 h Fecal	0.060	0.060
Vagina	7 d Fecal	0.800	0.800
Vagina	42 d Fecal	0.999	0.800
Vagina	60 d Fecal	0.700	0.700
Oral	Cow Fecal	0.020	0.020
Oral	Meconium	0.020	0.020
Oral	24 h Fecal	0.004	0.004
Oral	7 d Fecal	0.100	0.100
Oral	42 d Fecal	0.300	0.300
Oral	60 d Fecal	0.400	0.400
Cow Fecal	Meconium	0.900	0.700
Cow Fecal	24 h Fecal	0.004	0.004
Cow Fecal	7 d Fecal	0.004	0.004
Cow Fecal	42 d Fecal	0.004	0.004
Cow Fecal	60 d Fecal	0.004	0.004
Meconium	24 h Fecal	0.004	0.004
Meconium	7 d Fecal	0.004	0.004
Meconium	42 d Fecal	0.004	0.004
Meconium	60 d Fecal	0.004	0.004
24 h Fecal	7 d Fecal	0.010	0.008

24 h Fecal	42 d Fecal	0.004	0.004
24 h Fecal	60 d Fecal	0.004	0.004
7 d Fecal	42 d Fecal	0.050	0.050
7 d Fecal	60 d Fecal	0.020	0.020
42 d Fecal	60 d Fecal	0.800	0.800

[1]A *P*-value ≤ 0.05 was considered significant.

Table S2 Permutational Analysis of Variance (PERMANOVA) pairwise comparison results based on sample type using Weighted Unifrac distances.

Sample Type 1	Sample Type 2	Pseudo-f statistic	Bonferonni *P* – value[1]
Placenta	Colostrum	2.071	0.999
Placenta	Vagina	6.093	0.714
Placenta	Oral	10.224	0.097
Placenta	Cow Fecal	11.906	0.097
Placenta	Meconium	6.408	0.097
Placenta	24 h Fecal	16.495	0.097
Placenta	7 d Fecal	9.981	0.097
Placenta	42 d Fecal	9.601	0.097
Placenta	60 d Fecal	10.572	0.097
Colostrum	Vagina	4.702	0.214
Colostrum	Oral	7.883	0.097
Colostrum	Cow Fecal	9.284	0.097
Colostrum	Meconium	6.140	0.097
Colostrum	24 h Fecal	10.747	0.097
Colostrum	7 d Fecal	7.659	0.097
Colostrum	42 d Fecal	7.418	0.097
Colostrum	60 d Fecal	8.084	0.097
Vagina	Oral	3.194	0.999
Vagina	Cow Fecal	4.611	0.214
Vagina	Meconium	2.445	0.999
Vagina	24 h Fecal	12.008	0.214
Vagina	7 d Fecal	3.625	0.999
Vagina	42 d Fecal	2.424	0.999
Vagina	60 d Fecal	3.016	0.643
Oral	Cow Fecal	14.984	0.097
Oral	Meconium	6.932	0.195
Oral	24 h Fecal	6.953	0.097
Oral	7 d Fecal	3.105	0.999
Oral	42 d Fecal	4.535	0.999
Oral	60 d Fecal	6.109	0.584
Cow Fecal	Meconium	2.085	0.999
Cow Fecal	24 h Fecal	77.318	0.097
Cow Fecal	7 d Fecal	14.099	0.097
Cow Fecal	42 d Fecal	9.707	0.097
Cow Fecal	60 d Fecal	8.474	0.097
Meconium	24 h Fecal	21.121	0.097
Meconium	7 d Fecal	6.941	0.097
Meconium	42 d Fecal	4.252	0.097
Meconium	60 d Fecal	3.623	0.195
24 h Fecal	7 d Fecal	9.944	0.097
24 h Fecal	42 d Fecal	17.294	0.097

24 h Fecal	60 d Fecal	26.499	0.097
7 d Fecal	42 d Fecal	3.221	0.974
7 d Fecal	60 d Fecal	4.998	0.195
42 d Fecal	60 d Fecal	0.857	0.999

[1]A Bonferroni corrected *P*-value ≤ 0.05 was considered significant.

Table S3 Relative abundance of phyla within microbiome of each sample type from multiparous Holstein cows (n = 6) and their calves (n =6; bulls = 3, heifers =3).

Phylum	Placenta	Colostrum	Vagina[1]	Oral	Cow Fecal	Meconium	24 h Fecal	7 d Fecal	42 d Fecal	60 d Fecal
Crenarchaeota	0.000	0.000	0.000	0.000	0.000	0.005	0.000	0.000	0.000	1.560E-4
Euryarchaeota	0.414	0.000	0.114	0.059	1.025	0.232	0.000	0.000	0.024	0.232
{Unknown Phylum} Bacteria-1	0.000	0.000	0.001	1.100E-4	3.360E-4	0.000	0.000	0.000	0.000	0.000
{Unknown Phylum} Bacteria-2	0.000	0.000	0.001	4.380E-4	0.009	0.002	0.000	0.000	0.000	0.000
[Thermi]	0.000	0.000	0.016	0.021	0.002	0.006	0.000	0.000	0.000	0.000
Acidobacteria	0.000	0.000	0.006	0.008	0.005	0.061	0.000	1.000E-4	2.870E-4	1.560E-4
Actinobacteria	19.729	2.049	0.476	1.150	1.280	1.149	0.003	6.500	0.914	0.487
Armatimonadetes	0.000	0.000	0.000	0.000	0.000	0.004	0.000	0.000	0.000	0.000
Bacteroidetes	15.510	1.177	8.057	16.013	48.812	42.554	0.043	43.362	49.352	45.575
BRC1	0.000	0.000	0.000	0.001	0.000	0.001	0.000	0.000	0.000	0.000
Chlamydiae	0.000	0.000	0.000	3.290E-4	0.000	0.000	0.000	0.000	0.000	0.000
Chlorobi	0.000	0.000	4.940E-4	0.001	0.000	0.000	0.000	0.000	0.000	0.000
Chloroflexi	0.000	0.000	0.017	0.020	0.005	0.094	7.050E-5	2.010E-4	0.001	0.001
Cyanobacteria	0.050	0.000	0.119	0.213	0.969	0.585	7.050E-5	2.010E-4	0.753	0.257
Deferribacteres	0.000	0.000	0.000	0.000	0.003	0.000	0.000	0.000	0.000	0.000
Elusimicrobia	0.000	0.000	0.000	0.001	0.030	0.002	0.000	1.000E-4	0.000	0.253
FBP	0.000	0.000	3.290E-4	0.001	0.000	0.001	0.000	0.000	0.000	0.000
Fibrobacteres	0.000	0.000	0.003	0.010	0.048	0.128	0.000	0.000	0.000	0.000
Firmicutes	15.961	0.599	20.043	10.504	40.153	20.502	14.847	27.153	24.050	32.975
Fusobacteria	0.013	0.000	0.317	0.191	0.001	0.003	0.001	3.830	0.127	0.007
Gemmatimonadetes	0.000	0.000	0.003	0.002	0.000	0.022	0.000	0.000	0.000	0.000
GN02	0.000	0.000	0.001	0.012	0.000	0.000	0.000	0.000	0.000	0.000
GN04	0.000	0.000	0.000	1.100E-4	0.000	0.000	0.000	0.000	0.000	0.000
LD1	0.000	0.000	0.000	0.000	0.001	0.000	0.000	0.000	0.000	0.000
Lentisphaerae	0.000	0.000	0.063	0.078	0.145	0.127	2.470E-4	1.510E-4	0.000	0.003
Nitrospirae	0.000	0.000	0.000	1.100E-4	0.001	0.014	7.050E-5	0.000	0.000	0.000
NKB19	0.000	0.000	0.000	3.290E-4	3.360E-4	0.001	0.000	0.000	0.000	0.000
OD1	0.000	0.000	0.002	0.003	0.000	0.012	0.000	0.000	0.000	0.000
OP11	0.000	0.000	0.001	0.002	0.000	0.000	0.000	0.000	0.000	0.000
Planctomycetes	0.006	0.000	0.036	0.032	0.067	0.081	1.060E-4	0.000	0.000	0.000
Proteobacteria	47.702	96.153	57.843	69.330	2.373	30.859	85.104	19.144	23.902	11.327
Spirochaetes	0.233	0.011	0.050	0.179	1.355	0.627	4.580E-4	0.001	0.002	8.320
Synergistetes	0.000	0.000	0.000	0.000	0.002	0.000	0.000	0.000	0.000	0.001

Tenericutes	0.276	0.000	12.147	1.777	0.436	0.921	0.001	3.010E-4	0.875	0.562
TM6	0.000	0.000	0.000	1.640E-4	0.000	0.000	0.000	0.000	0.000	0.000
TM7	0.000	0.000	0.000	0.001	0.000	0.001	0.000	0.000	0.000	0.000
Verrucomicrobia	0.107	0.011	0.681	0.388	3.279	2.006	3.530E-4	0.009	0.000	1.560E-4
WPS-2	0.000	0.000	0.002	0.001	0.000	0.001	0.000	0.000	0.000	0.000
WWE1	0.000	0.000	0.000	1.640E-4	0.001	0.000	0.000	0.000	0.000	0.000

[1]Vaginal canal samples were only obtained from 4 Holstein cow-calf pairs.
